# Olfactory bulb volume changes associated with trans-sphenoidal pituitary surgery

**DOI:** 10.1371/journal.pone.0224594

**Published:** 2019-12-18

**Authors:** Dino Podlesek, Amir Zolal, Matthias Kirsch, Gabriele Schackert, Thomas Pinzer, Thomas Hummel

**Affiliations:** 1 Department of Neurosurgery, Dresden University of Technology, Carl Gustav Carus Faculty of Medicine, Dresden, Germany; 2 Department of Spine Surgery and Neurotraumatology, SRH-Wald Clinic Gera, Gera, Germany; 3 Department of Neurosurgery, Asklepios Clinic Schildautal Seesen, Germany; 4 Interdisciplinary Smell & Taste Clinic, Department of Otorhinolaryngology, Dresden University of Technology, Carl Gustav Carus Faculty of Medicine, Dresden, Germany; Universitatsklinikum Freiburg, GERMANY

## Abstract

**Objective:**

The trans-sphenoidal approach is most frequently used for pituitary adenoma (PA) enucleation. However, effects of this surgery on neighboring structures have received little attention so far. In particular, no investigations on olfactory bulb (OB) anatomy after trans-sphenoidal surgery have been reported. Because impairment of olfaction has been shown in small groups following trans-sphenoidal surgery we hypothesized that the transnasal approach is likely to alter OB volume which is associated with changes of olfactory function.

**Methods:**

The study comprised 33 patients with pituitary adenoma (14 women and 19 men, mean age 50 years). Comprehensive assessment of olfactory function was conducted with the "Sniffin' Sticks" test kit. Based on magnetic resonance imaging scans OBs were measured before and approximately one year after trans-sphenoidal PA enucleation.

**Results:**

Owing to postoperative non-compliance and MRI artifacts partly due to drill friction complete evaluation of “Sniffin' Sticks” in term of obtaining the TDI score was possible pre- and postoperatively in 21 patients whereas OB volumes were available in 32 patients. Approximately one year after surgery olfactory function was not significantly different from baseline. However, left- and right-sided OB volume in patients treated via trans-sphenoidal surgery decreased (p = 0.001). The side of the surgical approach did not affect OB volume in a side-specific manner. Changes in odor threshold were significantly correlated to changes in right-sided OB volume (r = 0.45, p = 0.024).

**Conclusion:**

Overall olfactory performance one year after surgery was not significantly different from baseline. However, changes in OB volume are associated with changes in olfactory performance and OB volumes decreased in patients.

## Introduction

Transnasal trans-sphenoidal pituitary surgery is a common and well established approach for removing lesions from the sellar region with 10–15% of all brain tumors being related to this area [1]. Main goals are total enucleation of the tumor, preservation of the olfactory neuroepithelium, care of neuroendocrine and visual structures [2]. Due to the operative approach and its interference with the nasal mucosa numerous rhinological complications have been reported [3–11]. In addition, impairment of olfactory function after trans-sphenoidal surgery has been described [12–14]. However, there have been no investigations comparing the olfactory bulb (OB) volume after trans-sphenoidal surgery in patients with pituitary mass lesions.

Accordingly, the primary objective of our study was to evaluate OB volume in patients undergoing transnasal trans-sphenoidal surgery for pituitary adenoma (PA). Considering the neuroplasticity of the OB [15] and its response to changes in olfactory sensitivity we expected a volumetric change in this neuroanatomic structure.

## Materials and methods

The study was approved by the Ethics Committee of the Faculty of Medicine Carl Gustav Carus at the Technische Universität Dresden, Germany (EK435122011). The study was performed according to the Declaration of Helsinki. Written informed consent was obtained from each participant. Raw data of the study will be made available upon request.

Between April 2012 to May 2013 47 patients were operated on PA through transsphenoidal approach. Due to previous transcranial or transsphenoidal operations on PA or by reason of non-compliance and/or study refusal 14 patients did not meet inclusion criteria. Fourteen women and 19 men (mean age 50 years) scheduled to undergo trans-sphenoidal surgery for pituitary gland and sellar region tumor were included into our prospective study between April 2012 and May 2013. Pre- and postoperatively hormonal lab work-up and physical examination were performed. In all patients neurosurgery was performed via the microscopic transnasal, trans-sphenoidal approach. All patients were operated for the first time. There were no medical histories on craniocerebral injury or sinonasal disease that could have affected olfactory function. Unspecific headache, visual field impairment, adynamia, enlarged feet / facial bones / hands indicative of hormone disorders led to physical examination in outpatient clinics and consequently to additional diagnostic procedure in terms of cerebral MRI and referral to our department. In all patients intra/supra/parasellar tumors were identified in cranial MRI. Two of the patients had symptoms of acromegaly. One patient was diagnosed with Cushing´s disease. Preoperatively, in each patient otorhinolaryngological diseases were excluded by clinical examination and detailed medical history.

Olfactory testing: Validated “Sniffin' Sticks” tests were utilized for the psychophysical testing of olfactory function performed pre- and approximately 310 days postoperatively. Odors were presented to both nostrils in dispensers similar to felt-tip pens (“Sniffin' Sticks”, Burghart GmbH, Wedel, Germany) [16]. For each patient scores for odor threshold, odor discrimination, and odor identification were obtained [16, 17]. According to published norms, patients were diagnosed with normosmia, hyposmia and functional anosmia [18, 19].

Operative procedure: Dependent on lesion configuration an uninostril direct microsurgical technique to the anterior wall of the sphenoid sinus was selected. The configuration of the tumor was included into approach planning due to its slight diagonal trend. All patients were to undergo surgery in semi-sitting position. The right thigh was prepared for a potential preparation of fascia lata. Septum nasi was dislocated to the contralateral side. The mucosa of the anterior wall of the sphenoid sinus was divided in a blunt manner. Coagulation was rarely used. The mucosal layer of the anterior sphenoidal wall was dissected from medial to lateral of the nasal septum to prevent mucosal lesions. The inserted speculum was placed in front of the anterior wall of the sphenoid sinus. The mucosa of the anterior sphenoid wall was incised and scrapped sideways. Bony aspects of the anterior sphenoid wall were removed. After tumor removal the “mucosal blanket” was folded back to its place from lateral to medial to ensure its approximate anatomic position. It was fixated by applying intranasal tamponades. Throughout surgery care was taken to preserve the olfactory neuroepithelium.

**Fig 1:**
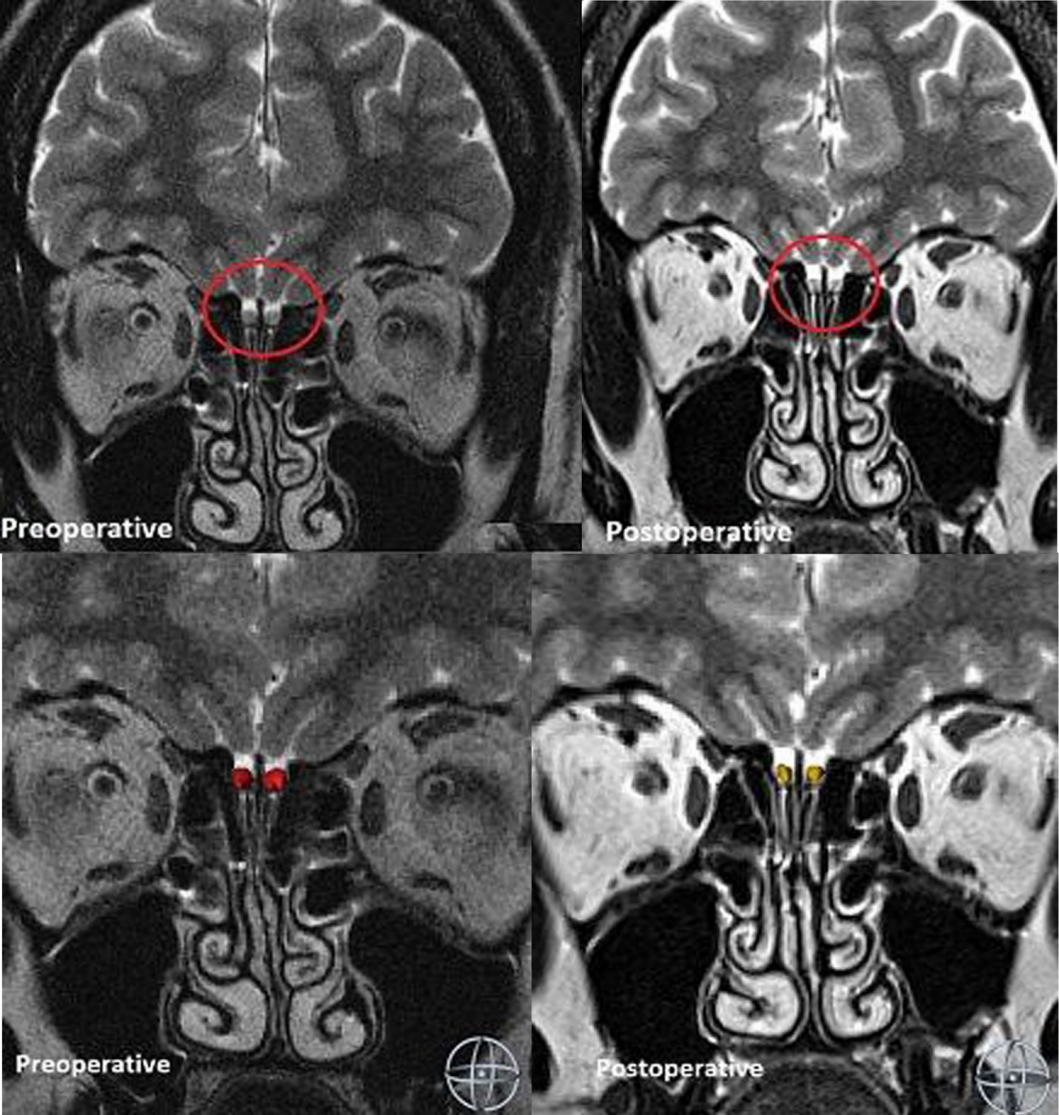
Segmented OB in a 60 years old female patient with pituicytoma pre- and postoperatively on the basis of T2 coronar images. Postoperative volumetry was performed 13 months after transsphenoidal tumor enucleation. The protocol included T2-weighted 2D turbo spin echo (TSE) sequence in the coronal plane (matrix 256×256; field of view 120×120; slice thickness 2.0 mm; number of slices 30). Olfactory bulbs were smaller postoperatively.

OB MRI: In addition to standard pituitary neuro-imaging supplementary MRI images of OBs were generated in all patients. Coronal sequences exposing the OB in its groove along the frontal skull base were used for measurements; they were obtained with a 1.5 Tesla magnetic resonance imaging system (Sonata; Siemens, Erlangen, Germany). The protocol included T2-weighted 2D turbo spin echo (TSE) sequence in the coronal plane (repetition time [TR] = 4800 ms; echo time [TE] = 152 ms; flip angle, 150°; number of averages = 4; matrix 256×256; field of view 120×120; slice thickness 2.0 mm; number of slices 30). Both OBs were measured separately, by neurosurgeon (not involved in operative procedure) blinded to the olfactory outcome, using AMIRA 3D visualization software (Visage Imaging, Carlsbad, USA) (Fig 1).

Statistical analyses: All statistics were performed using SPSS software version 23.0 (SPSS Inc., Chicago, IL, USA). For paired samples (pre-/postoperatively comparison) t-tests were utilized. When correlating OB volumes with TDI test scores, the differences post-/pre-surgery were used for TDI scores and OB volumes. Spearman statistics were used for this approach; coefficients of correlations are presented with the number of analyzed subjects in subscript. The level of significance was set at 0.05.

## Results

Owing to postoperative non-compliance and MRI artifacts due to drill friction complete evaluation of “Sniffin' Sticks” in term of obtaining the TDI score was possible preoperatively in 28 patients and postoperatively in 25 patients, although odor thresholds and odor identification could be obtained in slightly larger subsets of patients (Table 1); these differences were largely due to the compliance of patients with the olfactory tests. Volumetric measurements of the OB were obtained in 32 patients (Table 1).

**Table 1:** Olfactory-related scores (means, standard deviations) before and after surgery. For comparability results are only listed for those patients who had complete measurements before and after surgery. The two columns on the right indicate results from correlation analyses made between measures obtained before and after surgery and the results from tests for differences (t-tests) between measures before and after surgery.

		Before surgery		After surgery		Correlation between measures obtained before and after surgery	Differences between measures before and after surgery
	N	Mean	SD	Mean	SD		
OB right	32	48.2	15.9	42.7	12.4	r = 0.85, p<0.001	t = 3.84, p = 0.001
OB left	32	47.1	18.4	40.9	15.9	r = 0.81, p<0.001	t = 3.69, p = 0.001
TDI score	21	31.1	4.4	30.5	5.4	r = 0.18, p = 0.43	t = 0.41, p = 0.69
Odor threshold	26	6.2	2.8	5.7	2.5	r = 0.45, p = 0.02	t = 0.80, p = 0.43
Odor discrimination	21	11.7	2.0	11.3	2.7	r = 0.19, p = 0.42	t = 0.53, p = 0.60
Odor identification	25	13.6	1.5	13.2	1.6	r = -0.-17, p = 0.42	t = 0.79, p = 0.44

Although 28 patients provided a TDI score before surgery and 25 patients provided a TDI score after surgery, only twenty-one patients provided a complete TDI score both before and after surgery. Of those 21 patients with complete TDI olfactory scores before surgery 10 were classified as hyposmic, and 11 as normosmic. After surgery one patient was classified as functionally anosmic, 8 as hyposmic, and 12 patients as normosmic.

One year after surgery olfactory function was not significantly different from baseline (p>0.40). With regard to odor thresholds 7 patients decreased in function by 2.5 points or more [20].

**Fig 2:**
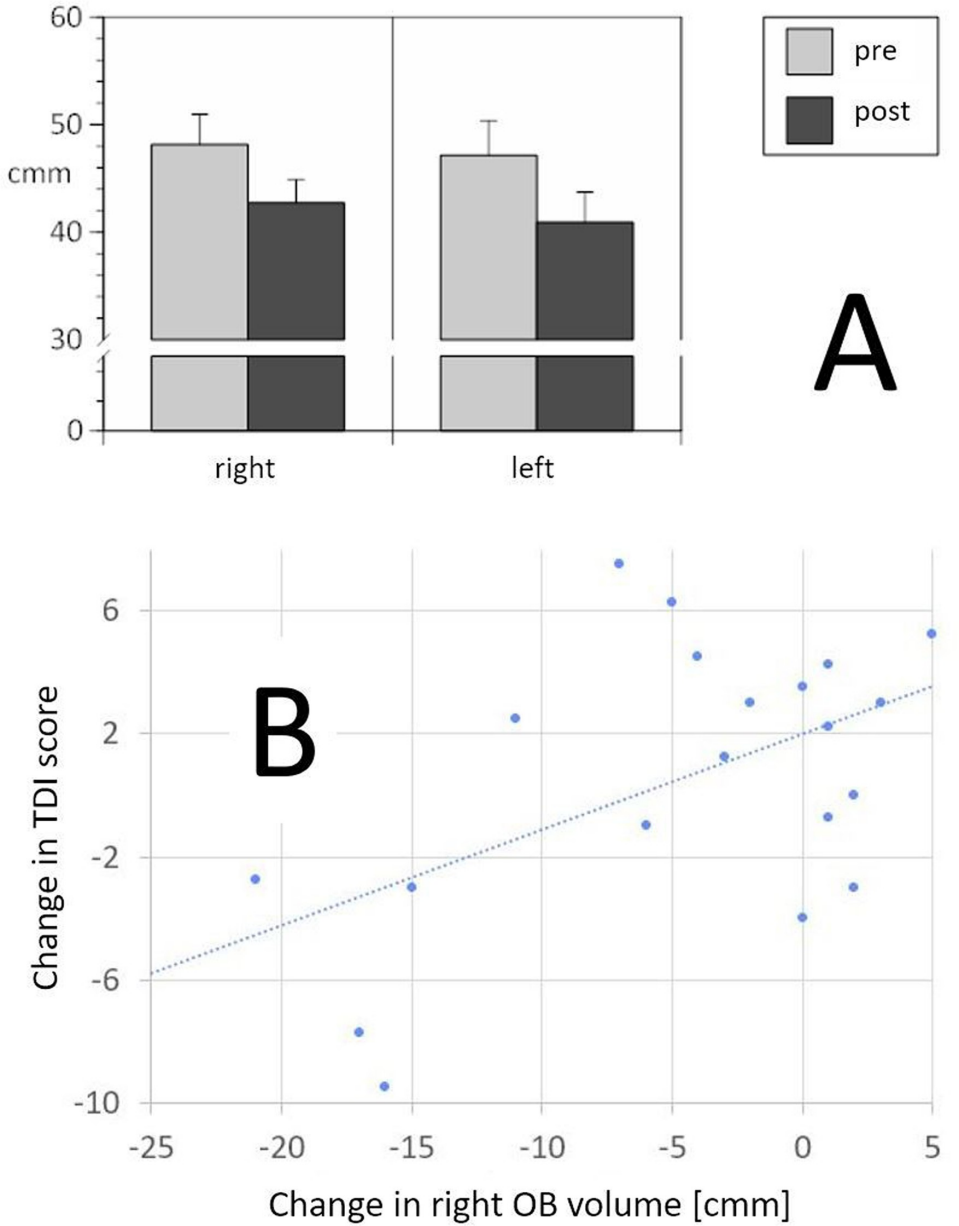
**A**: OB volume (means, SEM) before and after surgery, separately for the left and right side; **B**: Correlation between the change of the right-sided OB volume and TDI score (r_**20**_ = 0.52, p = 0.018; for this display an outlier was removed–with the outlier the coefficient of correlation is slightly smaller: r_**21**_ = 0.48, p = 0.026). The graph shows that in 4 patients a slightly improved olfactory function is associated with an increased right-sided OB volume. The gravity of the changes, however, is on the side of smaller olfactory bulbs accompanied by decreased olfactory function.

At the time of the second measurement (on average 302 days [range 175–562 days] after baseline measurement) OB volume in patients treated via trans-sphenoidal surgery was significantly smaller (p = 0.001; right side: mean difference 5.4mm^3^; 95%CI 2.6–8.3 mm^3^; left side: mean difference 6.2 mm^3^ 95%CI 2.8–9.7 mm^3^) (Fig 2A) (Table 1).

When using the normative values of OB volume investigated in a large cohort by Buschhuter et al. as a reference [21], 16 patients exhibited a relatively small OB volume already before surgery. However, the number of relatively small OBs increased to 28 after surgery.

In comparison to pre-surgical measurements, after surgery a decreased OB volume was found for the left side in 22 patients, in 3 patients it stayed the same, and in 7 patients it increased; for the right OB these numbers were 21 (decrease), 2 (same), and 9 (increase).

Changes in odor threshold, but not changes in odor identification or odor discrimination, were positively correlated with changes in right-sided OB volume (r_25_ = 0.45, p = 0.024), indicating that postoperative decrease in olfactory sensitivity was associated with postoperative decrease in right-sided OB volumes (see Fig 2B). Interestingly, the side of the surgical approach did not affect OB volume in a side-specific manner.

## Discussion

Major results of the current study were that approximately one year after surgery (1) olfactory function was not significantly different from baseline; however, (2) OB volume in patients treated via trans-sphenoidal surgery decreased. (3) These changes in OB volume tended to correlate with changes in odor thresholds (Table 1).

Surgical manipulation and impairment of the olfactory pathways are often mirrored in the structural change of OBs [4, 22]. Measuring the OB with MR images is not part of daily clinical routine. Yet it is easily carried out, reliable and provides a reproducible measure of olfactory function [23] which was indicated by the correlation between changes of odor thresholds and changes in OB volume.

The olfactory system and the dentate gyrus of the hippocampus have the ability to neurogenesis in adulthood [24, 25]. Although there is ongoing neurogenesis in the adult OB, only a part of newly generated neurons persevere [26]. The interaction between the olfactory epithelium and OB still harbors many questions. The olfactory epithelium has an impact on OB alteration and its neurogenesis can be influenced by the impairment of olfactory mucosa [27–29]. Olfactory neurons interact in the nasal cavity with the external milieu by olfactory dendrites [30]. The sensory neurons are activated by environmental chemosensory input and transfer this activation to the OBs [31]. In lack of trophic aid and synaptic contribution the epithelial neuronal progenitor cells do not develop further or rather perish [32]. Odor exposure increases survival of olfactory neurons [33]. In 2015 Nie et al. proposed an endoscopic endonasal trans-sphenoidal sub-septum mucosal approach for treating pituitary adenoma as an alternative approach for preserving and not traumatizing the olfactory mucosa [34]. Although the authors did not specify the olfactory tests and the follow up window, in their patient population (n = 52) all nasal mucosa was preserved and no “loss of smell” was present postoperatively. After the removal of intranasal tamponades none of the study patients reported smell impairment. Considering the low reliability of olfactory self-ratings these results by Nie et al. appear difficult to interpret. In fact, data on postoperative olfaction ability vary [4, 5, 8, 22, 35–38]. Slight differences in surgical management, different smell tests and clinical follow up in variable intervals are potential causes for the diversity of these results. Rioja et al. (2015) reported that olfactory function declined after 12 months in their group of patients after transnasal trans-sphenoidal endoscopic approach and expanded endonasal approach [35]. In contrast, Hart et al. reported no significant change of olfactory function in the majority of their patients three months after surgery which included the resection of the posterior aspect of the septum and therewith loss of a part of olfactory neuroepithelium.

Minimally invasive trans-sphenoidal route requires a small speculum to be inserted through the nasal cavity straight to the anterior wall of the sphenoid sinus. On this way surgery interferes with the medial turbinate and anterior nasal septum. There are several ways how olfactory function could be affected. (1) The sensory receptors of the main olfactory system are located in the upper third of the nasal cavity starting from the insertion of the middle turbinate [39]. Accordingly, surgery might affect olfactory receptors located at the middle turbinate and the septum. (2) Sphenopalatine, facial and ophthalmic veins are a major venous drainage for the nasal mucosa. Surgery might affect that extensive network of blood vessels in the mucosa which could result in olfactory impairment. Finally, (3) surgery might change the distribution of intranasal airflow which is significantly related to the perception of odors [40, 41].

To investigate the question whether the currently investigated patents already exhibited an olfactory problem prior to surgery, we additionally performed a comparison between results from Buschhuter et al. [21] and the current sample. From the Buschhuter sample we selected a sample of 29 women and 28 men with an age between 33 and 79 years so that there was no significant difference between the two samples in terms of age (p = 0.50) and sex (p = 0.39). In fact, the currently investigated sample exhibited significantly smaller olfactory bulbs (p = 0.001) indicating compromised olfactory function prior to surgery. This emphasizes the thought that it is important to measure olfactory function prior to surgery in order to be able to evaluate possible postoperative complaints.

We are aware that our study has several limitations. Due to patient follow up in the pituitary outpatient clinic we were able to monitor those as recently as one year. It has been shown that endoscopic approaches to skull base are associated with short term olfaction impairment, although three months and one year after transsphenoidal surgery for pituitary lesions the overall olfactory function was not significantly different from baseline[42, 43]. The patient cohort was not large and olfactory testing was done birhinally. Besides we have tested olfactory function in an extensive manner using a validated and reliable tool. Prospective examinations should imply the inspection of the paranasal sinuses and additional short-term examination follow up. Although none of our patients reported a major decrease of olfactory function in long term follow up, short-term tests could possibly reveal pronounced impairment of olfactory function after trans-sphenoidal approach as shown by several authors [4, 35, 36]. It should also be kept in mind that half of the patients had already a decreased OB volume before surgery. Therefore, it seems possible that a pituitary adenoma itself is associated with reduced OB volume. This should be clarified in future studies. Further, it could be assumed that the approximate one year interval between the two measurements of the OB by itself could have produced a significant difference in OB volume. Although it may have contributed to changes in OB structure, still, based on data from Buschhuter et al. for the right OB and age groups between 50 and 60 years such an effect would amount to an average decrease of 0.8 mm^3^/year which does not explain the present observed difference in OB volume of approximately 6 mm^3^ [21].

## Conclusion

Olfactory bulb volume after trans-sphenoidal transnasal pituitary surgery is significantly affected as indicated by changes in its volume.

## Supporting information

S1 Table S1 Table shows Olfactory Bulb volumetry / measurements ((olfactory bulb volume in cmm right/left; before and after surgery); side of surgical approach to pituitary gland (L left; R right), age in years, gender (male, female), Olfactory performance assessed by the Combined Testing of Odor Identification, Odor Discrimination and Olfactory Threshold—Sniffin' Sticks (TDI-Score (T threshold score, D discrimination score, I identification score; before and after surgery), Interval between measurements (before and after surgery) in days).
(XLSX)
